# Differences in *EGFR* and *KRAS* mutation spectra in lung adenocarcinoma of never and heavy smokers

**DOI:** 10.3892/ol.2013.1551

**Published:** 2013-08-29

**Authors:** KAZUYA TAKAMOCHI, SHIAKI OH, KENJI SUZUKI

**Affiliations:** Department of General Thoracic Surgery, Juntendo University School of Medicine, Bunkyo-ku, Tokyo 113-8431, Japan

**Keywords:** lung cancer, adenocarcinoma, smoking, epidermal growth factor receptor, *KRAS*, mutation

## Abstract

Epidermal growth factor receptor (*EGFR*) mutations are common in lung adenocarcinomas of never smokers, while *KRAS* mutations are more frequent among heavy smokers. Different clinicopathological and biological characteristics may, therefore, exist in lung adenocarcinoma according to smoking status. In the present study, a retrospective review was performed using 521 patients with surgically resected lung adenocarcinomas. The clinicopathological factors of age, gender, pathological tumor size, nodal status, lymphatic permeation and blood vessel invasion and the *EGFR* and *KRAS* mutation spectra were compared between never and heavy smokers. *EGFR* mutations were detected in 233 (45%) patients, while *KRAS* mutations were detected in 56 (11%) patients. *EGFR*-mutated adenocarcinomas had a higher prevalence of females in the never smokers compared with the heavy smokers (P<0.001). *KRAS*-mutated adenocarcinomas had a higher prevalence of females (P<0.001) and showed less frequent vascular invasion (P=0.018) in the never smokers compared with the heavy smokers. Minor *EGFR* mutations, excluding exon 21 L858R and exon 19 deletions, were more common in heavy smokers than never smokers (P=0.055). *KRAS* G to A transition was more common in never smokers, while *KRAS* G to T and G to C transversions were more common in heavy smokers (P=0.036). The clinicopathological characteristics and the spectra of the *EGFR* and *KRAS* mutations in lung adenocarcinoma were different between the never and heavy smokers. Further large-scale studies are required to evaluate the efficacy of molecular targeting agents with consideration to specific *EGFR* and *KRAS* mutations.

## Introduction

In lung cancer, epidermal growth factor receptor (*EGFR*) and *KRAS* mutations occur frequently in adenocarcinoma, rarely in squamous cell carcinoma and hardly ever in small cell carcinoma (1–3). These two mutations are known to have different correlations with smoking exposure. Specifically, *EGFR*-mutated adenocarcinomas are more common in individuals who have never smoked (1,3), while *KRAS*-mutated adenocarcinomas occur more frequently among heavy smokers (4). Therefore, we hypothesized that there may be different biological characteristics and etiologies between these types of lung adenocarcinomas related to cumulative exposure to tobacco smoke.

The presence of an *EGFR* mutation is the most important predictor of the efficacy of EGFR tyrosine kinase inhibitors (TKIs), such as gefitinib and erlotinib (5,6). Activating mutations in the TK domain of *EGFR* are limited to exons 18–21, but show marked structural diversity, including point mutations, deletions and insertions (1,3,5,6). The L858R in exon 21 and small deletions in exon 19 have been shown to account for 80–90% of all *EGFR* mutations and are often termed as classic activating mutations (3). Although an inverse correlation between the extent of smoking and the frequency of these mutations has been shown by a number of investigators (7,8), the correlation between smoking exposure and other minor mutations remains unclear.

*KRAS*-mutated lung adenocarcinomas are more predominant in Caucasians (~30%) than East Asians (~10%) (1). These mutations serve as a useful biomarker of resistance to EGFR-TKIs (9), occurring frequently at codon 12 and occasionally at codon 13, but rarely located at codon 61 (2). Several organ-specific *KRAS* mutation genotypes have been reported. The majority are G to T transversions in lung adenocarcinoma and G to A transitions in colorectal cancer (10). Although there have been few studies investigating the correlation between smoking exposure and *KRAS* mutation genotypes, Miller *et al*(11) reported that never smokers were significantly more likely than former or current smokers to have G to A transition mutations than G to T or G to C transversion mutations.

The purpose of the present study was to elucidate the differences in the clinicopathological characteristics of *EGFR*- and *KRAS*-mutated lung adenocarcinomas and their related mutation spectra between never smokers and heavy smokers.

## Materials and methods

### Patients

Between February 2009 and March 2012, 667 patients with primary lung cancer underwent pulmonary resection. Among these, 521 patients with adenocarcinoma were selected for retrospective review and examined for *EGFR* and *KRAS* mutations using surgically resected specimens. There were 277 (53%) never smokers (≤5 pack-years), 53 (10%) light smokers (5–20 pack-years) and 191 (37%) heavy smokers (>20 pack-years). Induction chemotherapy, radiotherapy and pre-operative treatment with EGFR-TKIs were not performed for any of the patients in this series.

This study was conducted on specimens stored in the tissue bank, with the approval of the Institutional Review Board (IRB) of Juntendo University School of Medicine (Tokyo, Japan). According to the tissue bank protocol, in order to collect specimens for studies gaining approval by the IRB in the future, written consent was obtained from patients prior to surgery for the collection and storage of specimens during surgery. The contents of this study were deemed ethically acceptable and the IRB approved the use of the specimens stored in the tissue bank without obtaining new informed consent.

### Molecular analysis

Genomic DNA was extracted from 3–5-mm^3^ cubes of frozen fresh lung cancer tissue samples from surgically resected specimens. The peptide nucleic acid-locked nucleic acid (PNA-LNA) polymerase chain reaction (PCR) clamp method (12) was used to identify *EGFR* mutations: G719A, G719C and G719A in exon 18; all deletion genotypes in exon 19; T790M in exon 20; and L858R and L861Q in exon 21. The PNA-mediated PCR clamping method (13) was used to identify *KRAS* mutations: All genotypes at codon 12 and 13. Molecular analyses for the *EGFR* and *KRAS* mutations were conducted at Mitsubishi Chemical Medience Corporation (Tokyo, Japan).

### Statistical analysis

The clinicopathological factors of age, gender, pathological tumor size, nodal status, lymphatic permeation and blood vessel invasion were compared between the never and heavy smokers, along with the *EGFR* and *KRAS* mutation spectra. A χ^2^ test, Fisher’s exact test or t-test was used for the statistical analysis. P<0.05 was considered to indicate a statistically significant difference. All statistical analyses were performed using the SPSS statistical software package (version 20.0; SPSS, Inc., Chicago, IL, USA).

## Results

### EGFR and KRAS mutations in all adenocarcinoma patients

*EGFR* and *KRAS* mutations were detected in 233 (45%) and 56 (11%) of the total 521 lung adenocarcinoma patients, respectively. These two mutations were mutually exclusive. The point mutation L858R in exon 21 and deletions in exon 19 were detected in 118 and 98 tumors, respectively, and together, they accounted for 93% of all *EGFR* alterations. The remaining minor *EGFR* mutations included exon 18 G719A in eight tumors, exon 18 G719S in three tumors, exon 18 G719C in three tumors, exon 21 L861Q in four tumors and exon 20 T790M in four tumors. Double mutations were identified in four tumors: Exon 21 L858R and exon 19 deletion in one tumor, exon 21 L861Q and exon 20 T790M in one tumor and exon 21 L858R and exon 20 T790M in two tumors. Notably, exon 20 T790M, which has been recognized as a mutation that confers resistance to EGFR-TKIs, was always coupled with other point mutations. With regard to *KRAS*, point mutations in codon 12 were observed in 54 (96%) tumors, and point mutations in codon 13 were detected in two (4%) tumors. G to T or G to C transversions were identified in 43 (77%) tumors, and G to A transition was observed in 13 (23%) tumors.

The clinicopathological characteristics based on *EGFR* and *KRAS* mutation status are summarized in Table I. *EGFR*-mutated adenocarcinomas were more common in female never smokers than *KRAS* mutations.

### Differences in characteristics of EGFR- and KRAS-mutated lung adenocarcinomas between never and heavy smokers

*EGFR* mutations were detected in 155 (67%) never smokers, 25 (11%) light smokers and 53 (23%) heavy smokers (Table II). *EGFR*-mutated adenocarcinomas had a higher female prevalence in never smokers compared with that in heavy smokers (P<0.001). Minor *EGFR* mutations, which exclude exon 21 L858R and exon 19 deletions, were more frequent in heavy smokers than in never smokers. However, this difference was not statistically significant (P=0.055).

*KRAS* mutations were detected in 11 (20%) never smokers, 8 (14%) light smokers and 37 (66%) heavy smokers (Table III). *KRAS*-mutated adenocarcinomas had a higher prevalence in females (P<0.001) and showed less frequent vascular invasion (P=0.018) in never smokers compared with heavy smokers. G to A transition was more common in never smokers, while G to T and G to C transversions were more common in heavy smokers (P=0.036).

### Gender differences in the characteristics of EGFR- and KRAS-mutated lung adenocarcinomas

There were no significant gender differences in age, pathological nodal status, lymphatic permeation, blood vessel invasion or mutation spectra for either *EGFR*- or *KRAS*-mutated lung adenocarcinomas (Tables IV and V). The mean tumor size of the *EGFR*-mutated adenocarcinomas was significantly larger in males compared with females (P=0.027; Table IV). However, the corresponding gender difference was not significant for *KRAS*-mutated adenocarcinoma (P=0.802; Table V).

## Discussion

Tobacco smoking is the main cause of lung cancer worldwide. Despite this, ~25% of worldwide (14) and 30–40% of Asian lung cancer patients (15) have never smoked. The occurrence of *EGFR* and *KRAS* mutations has been reported to be associated with smoking status. *EGFR* mutations are more commonly identified in the adenocarcinomas of never smokers (1,3), while *KRAS* mutations are more frequent in heavy smokers (4). Although no carcinogens causing *EGFR* or *KRAS* mutations in never smokers have yet been identified, the causes of each are suspected to differ based on smoking status. The present study showed notable differences in the clinicopathological characteristics and mutation spectra of lung adenocarcinoma based on cumulative exposure to tobacco smoke.

It is noteworthy that minor *EGFR* mutations, which exclude exon 21 L858R and exon 19 deletions, tended to be more common in heavy smokers than in never smokers. However, it remains unclear why the incidence of exon 21 L858R and exon 19 deletions is so much higher than the various other TK domain mutations of *EGFR*(1,3). There should be specific mutagens (other than those associated with smoking) associated with these classic activating *EGFR* mutations. By contrast, the minor mutations of *EGFR* may randomly occur due to carcinogens present in tobacco smoke.

The correlation between EGFR-TKI efficacy and minor mutations is not fully clear. An *in vitro* study showed that gefitinib had variable growth-suppressive effects for different *EGFR* mutant-expressing cells (16). In a previous study of 62 lung cancer patients with minor *EGFR* mutations, Wu *et al*(17) observed a favorable response to EGFR-TKIs in 28 patients with point mutations on G719 and L861 and a poor response in 34 patients with different mutations. De Pas *et al*(18) also observed different antitumor activities of EGFR-TKIs according to the specific *EGFR* mutation genotype in a study of 10 lung cancer patients with minor mutations. The efficacy of EGFR-TKI treatment in patients with minor mutations is diverse and depends on the specific *EGFR* mutation.

*KRAS* and *TP53* mutations in lung cancer have been reported to occur more frequently in smokers than in never smokers (4,19). Fewer studies have investigated mutation spectrum variations for *KRAS* according to smoking status than *TP53* mutations, yet similar correlations between the two have been reported (19–21). Specifically, G to T transversion is most common in lung cancers in smokers, while G to A transition is more frequent among never smokers. Riely *et al*(21) demonstrated that not only G to T, but also G to C transversion frequently occurs in smokers with *KRAS*-mutated lung adenocarcinomas. The results of the current study are in agreement with these previous observations.

The association between *KRAS* mutation and the lack of benefit of anti-EGFR monoclonal antibodies, such as cetuximab and panitumumab, in metastatic colorectal cancer has been established (22). By contrast, for advanced non-small cell lung cancer, the benefit of cetuximab has been shown regardless of *KRAS* mutation status in a phase III trial of cisplatin and vinorelbine treatment, with or without cetuximab (23,24). Notably, the *KRAS* mutation spectrum in colorectal cancer is quite different from that in lung cancer of smokers; G to T transversion is most common in lung cancer, while G to A transition is most common in colorectal cancer (10). The *KRAS* mutation spectrum may therefore be a better determinant of anti-EGFR monoclonal antibody efficacy than simply the presence or absence of the *KRAS* mutation. G to A transition is frequent not only in colorectal cancer, but also in lung cancer of never smokers, indicating that the same mutagen may be associated with their carcinogenic processes.

Gender differences in lung cancer susceptibility have been previously reported. Several studies have provided evidence for a biological role of estrogen in lung carcinogenesis by direct promotion of cell proliferation (25,26). Environmental risk factors including indoor air pollution from cooking-oil fumes (27) and coal burning (28) have been reportedly associated with high lung cancer incidence among Chinese women. However, in the present study, no gender differences were observed in the characteristics of the *EGFR*- or *KRAS*-mutated lung adenocarcinomas other than tumor size, indicating that smoking status has a greater effect on the cause of lung adenocarcinoma than gender differences in Japanese patients.

In conclusion, in the present study, the clinicopathological characteristics and the spectra of *EGFR* and *KRAS* mutations in lung adenocarcinoma were different between never and heavy smokers. These findings indicate that the causes of these mutations in lung adenocarcinomas are different in never and heavy smokers. The efficacy of molecular targeting agents may differ based on the mutation spectra of the targeted genes. Therefore, in the future, the efficacy of these agents should be evaluated based not only on the presence of the corresponding gene mutation in an individual tumor, but also on the specific mutation spectrum of each gene. Further large-scale studies are recommended to further investigate and validate these spectra.

## Figures and Tables

**Table I:** Clinicopathological characteristics according to *EGFR* and *KRAS* mutation status in patients with lung adenocarcinomas.

Characteristics	*EGFR/KRAS* wild-type (n=232)	*EGFR* mutant (n=233)	*KRAS* mutant (n=56)	P-value^a^
Age, years				0.213^b^
Mean (range)	64 (24–86)	67 (35–88)	69 (44–87)	
Gender, n (%)				<0.001^c^
Male	124 (53)	81 (35)	40 (71)	
Female	108 (47)	152 (65)	16 (29)	
Smoking status, n (%)				<0.001^c^
Never smoker	111 (48)	155 (67)	11 (20)	
Smoker	121 (52)	78 (33)	45 (80)	
Tumor size, mm				0.072^b^
Mean (range)	23 (2–85)	24 (5–115)	29 (7–105)	
Pathological nodal status, n (%)				0.257^c^
N0	180 (78)	194 (83)	43 (77)	
N1/N2	52 (22)	39 (17)	13 (23)	
Lymphatic permeation, n (%)				0.644^c^
Positive	84 (36)	74 (32)	16 (29)	
Negative	148 (64)	159 (68)	40 (71)	
Vascular invasion, n (%)				0.448^c^
Positive	84 (36)	71 (30)	20 (36)	
Negative	148 (64)	162 (70)	36 (64)	

a P-values were derived from a comparison between *EGFR* and *KRAS* mutants;

b t-test;

c χ^2^ test.

*EGFR*, epidermal growth factor receptor.

**Table II:** Patient characteristics for *EGFR*-mutated lung adenocarcinomas according to smoking status.

Characteristics	Never smoker (n=155)	Light smoker (n=25)	Heavy smoker (n=53)	P-value^a^
Age, years				0.912^b^
Mean (range)	67 (35–88)	66 (37–85)	67 (48–86)	
Gender, n (%)				<0.001^c^
Male	26 (17)	15 (60)	40 (75)	
Female	129 (83)	10 (40)	13 (25)	
Tumor size, mm				0.383^b^
Mean (range)	24 (5–115)	23 (7–68)	22 (5–60)	
Pathological nodal status, n (%)				0.972^c^
N0	129 (83)	21 (84)	44 (83)	
N1/N2	26 (17)	4 (16)	9 (17)	
Lymphatic permeation, n (%)				0.221^c^
Positive	52 (34)	9 (36)	13 (25)	
Negative	103 (67)	16 (64)	40 (75)	
Vascular invasion, n (%)				0.944^c^
Positive	46 (30)	9 (36)	16 (30)	
Negative	109 (70)	16 (64)	37 (70)	
*EGFR* mutation				
Exon 21 L858R, n (%)				0.633^c^
Positive	79 (51)	14 (56)	25 (47)	
Negative	76 (49)	11 (44)	28 (53)	
Exon 19 deletion, n (%)				0.535^c^
Positive	69 (45)	8 (32)	21 (40)	
Negative	86 (55)	17 (68)	32 (60)	
Minor mutations, n (%)				0.055^d^
Positive	8 (5)	3 (12)	7 (13)	
Negative	147 (95)	22 (88)	46 (87)	

a P-values were derived from a comparison between never and heavy smokers;

b t-test;

c χ^2^ test;

d Fisher’s exact test.

*EGFR*, epidermal growth factor receptor.

**Table III:** Patient characteristics for *KRAS*-mutated lung adenocarcinomas according to smoking status.

Characteristics	Never smoker (n=11)	Light smoker (n=8)	Heavy smoker (n=37)	P-value^a^
Age, years				0.140^b^
Mean (range)	72 (58–87)	68 (55–77)	68 (44–86)	
Gender, n (%)				<0.001^c^
Male	3 (27)	5 (62.5)	32 (86.5)	
Female	8 (73)	3 (37.5)	5 (13.5)	
Tumor size, mm				0.550^b^
Mean (range)	34 (9–75)	30 (12–35)	30 (7–105)	
Pathological nodal status, n (%)				0.563^c^
N0	8 (73)	7 (87.5)	28 (76)	
N1/N2	3 (27)	1 (12.5)	9 (24)	
Lymphatic permeation, n (%)				0.070^c^
Positive	1 (9)	1 (12.5)	14 (38)	
Negative	10 (91)	7 (87.5)	23 (62)	
Vascular invasion, n (%)				0.018^c^
Positive	1 (9)	1 (12.5)	18 (49)	
Negative	10 (91)	7 (87.5)	19 (51)	
*KRAS* mutation, n (%)				
Codon 12 or 13				0.590^c^
Codon 12	11 (100)	8 (100)	35 (95)	
Codon 13	0 (0)	0 (0)	2 (5)	
Mutation spectrum				0.036^c^
G to T/G to C	6 (55)	5 (62.5)	32 (86)	
G to A	5 (45)	3 (37.5)	5 (14)	

a P-values were derived from a comparison between never and heavy smokers;

b t-test;

c Fisher’s exact test.

**Table IV:** Patient characteristics for *EGFR*-mutated lung adenocarcinomas according to gender.

Characteristics	Male (n=81)	Female (n=152)	P-value
Age, years			0.179^a^
Mean (range)	66 (37–86)	68 (35–88)	
Tumor size, mm			0.027^a^
Mean (range)	27 (5–115)	22 (5–70)	
Pathological nodal status, n (%)			0.595^b^
N0	66 (81)	128 (84)	
N1/N2	15 (19)	24 (16)	
Lymphatic permeation, n (%)			0.935^b^
Positive	26 (32)	48 (32)	
Negative	55 (68)	104 (68)	
Vascular invasion, n (%)			0.694^b^
Positive	26 (32)	45 (30)	
Negative	55 (68)	107 (70)	
*EGFR* mutation, n (%)			
Exon 21 L858R			0.779^b^
Positive	40 (49)	78 (51)	
Negative	41 (51)	74 (49)	
Exon 19 deletion			0.590^b^
Positive	36 (44)	62 (41)	
Negative	45 (56)	90 (59)	
Minor mutations			0.517^b^
Positive	5 (6)	13 (9)	
Negative	76 (94)	139 (91)	

a t-test;

b χ^2^ test.

*EGFR*, epidermal growth factor receptor.

**Table V:** Patient characteristics for *KRAS*-mutated lung adenocarcinomas according to gender.

Characteristics	Male (n=40)	Female (n=16)	P-value
Age, years			0.303^a^
Mean (range)	68 (44–86)	71 (57–87)	
Tumor size, mm			0.802^a^
Mean (range)	29 (7–105)	30 (7–75)	
Pathological nodal status, n (%)			0.548^b^
N0	31 (77.5)	12 (75)	
N1/N2	9 (22.5)	4 (25)	
Lymphatic permeation, n (%)			0.490^b^
Positive	12 (30)	4 (25)	
Negative	28 (70)	12 (75)	
Vascular invasion, n (%)			0.290^c^
Positive	16 (40)	4 (25)	
Negative	24 (60)	12 (75)	
*KRAS* mutation, n (%)			
Codon 12 or 13			0.506^b^
Codon 12	38 (95)	16 (100)	
Codon 13	2 (5)	0 (0)	
Mutation spectrum, n (%)			0.107^b^
G to T/G to C	33 (82.5)	10 (62.5)	
G to A	7 (17.5)	6 (37.5)	

a t-test;

b Fisher’s exact test;

c χ^2^ test.
